# Efficacy and safety of programmed cell death receptor 1 inhibition-based regimens in patients with pediatric malignancies: the real-world study in China

**DOI:** 10.3389/fimmu.2023.1182751

**Published:** 2023-06-09

**Authors:** Ye Hong, Mengjia Song, Yingxia Lan, Juan Wang, Suying Lu, Yu Zhang, Jia Zhu, Feifei Sun, Junting Huang, Juan Liu, Jiaqian Xu, Yanpeng Wu, Haixia Guo, Ruiqing Cai, Zijun Zhen, Yi Que, Yizhuo Zhang

**Affiliations:** ^1^ State Key Laboratory of Oncology in South China, Collaborative Innovation Center for Cancer Medicine, Sun Yat-sen University Cancer Center, Guangzhou, China; ^2^ Department of Pediatric Oncology, Sun Yat-sen University Cancer Center, Guangzhou, China; ^3^ Department of Pediatric, The Fifth Affiliated Hospital, Guangzhou Medical University, Guangzhou, Guang Dong, China; ^4^ Department of Pediatrics, Nanfang Hospital, Southern Medical University, Guangzhou, China

**Keywords:** pediatric malignancies, PD-1 inhibitors, PD-1 inhibitor-based treatment, safety, efficacy

## Abstract

**Background:**

Programmed death receptor 1 (PD-1) inhibition has shown durable response and mild adverse events (AEs) in adult malignancies. However, data on the clinical activity of PD-1 inhibition in pediatric patients are lacking. We comprehensively assessed the efficacy and safety of PD-1 inhibitor-based regimens for pediatric malignancies.

**Methods:**

We conducted a real-world, multi-institutional, retrospective analysis of pediatric malignancies treated with PD-1 inhibitor-based regimens. The primary endpoints were objective response rate (ORR) and progression-free survival (PFS). The secondary endpoints included disease control rate (DCR), duration of response (DOR), and AEs. The Kaplan–Meier method was used to calculate PFS and DOR. The National Cancer Institute Common Toxicity Criteria for AEs (version 5.0) were used to grade toxicity.

**Results:**

A total of 93 and 109 patients were evaluated for efficacy and safety, respectively. For all efficacy-evaluable patients, PD-1 inhibitor monotherapy, combined chemotherapy, combined histone deacetylase inhibitor, and combined vascular endothelial growth factor receptor tyrosine kinase inhibitor cohorts, the ORR and DCR were 53.76%/81.72%, 56.67%/83.33%, 54.00%/80.00%, 100.00%/100.00%, and 12.50%/75.00%, respectively; the median PFS and DOR were 17.6/31.2 months, not achieved/not achieved, 14.9/31.2 months, 17.6/14.9 months, and 3.7/1.8 months, respectively; the incidence rate of AEs were 83.49%, 55.26%, 100.00%, 80.00%, and 100.00%, respectively. One patient in the PD-1 inhibitor-combined chemotherapy cohort discontinued treatment due to diabetic ketoacidosis.

**Conclusions:**

This largest retrospective analysis demonstrate that PD-1 inhibitor-based regimens are potentially effective and tolerable in pediatric malignancies. Our findings provide references for future clinical trials and practice of PD-1 inhibitors in pediatric cancer patients.

## Introduction

Malignancies threaten children’s health with an estimated 121,145 cancer cases diagnosed among children and adolescents in China between 2018 and 2020. The world age-standardised incidence rates are 122.86 per million for children and 137.64 per million for adolescents (1). Through receiving intensive treatment, including surgery, chemotherapy, and radiotherapy, the prognosis of pediatric patients is generally favourable, and the 5-year survival rate has improved to 85–86% (2). However, intensive treatment is often accompanied by serious treatment-related adverse events (AEs), which severely affect the growth and quality of life of children and even lead to death (3–5). Besides, a subset of patients may present with refractory or relapsed disease, resulting in significant mortality (6). The 10-year progression-free survival (PFS) and overall survival from relapsed/progressive disease were only 18.4% ± 2.7% and 24.5% ± 3.0%, respectively (7). Therefore, there is an urgent need for novel therapeutic strategies with great curative effects and few AEs in pediatric malignancies.

Programmed death receptor 1 (PD-1) inhibitors have been approved for use in multiple adult cancers owing to their durable responses and mild AEs (8–10). However, there are few studies on the use of PD-1 inhibitors in children. Only the use of nivolumab, pembrolizumab, and atezolizumab monotherapy, nivolumab combined with ipilimumab, and nivolumab combined with metronomic cyclophosphamide have been reported in children with relapsed or refractory tumours, and the results suggested that PD-1 inhibitors are mainly effective in patients with lymphoma or germline DNA replication repair deficiency (11–16). There is little evidence regarding the application of PD-1 inhibitor-based combination therapies in pediatric malignancies, and multiple relevant clinical trials are still underway.

This retrospective study aimed to evaluate the efficacy and safety of PD-1 inhibitor-based regimens as first-line or salvage therapy for pediatric malignancies in the real world.

## Patients and methods

### Patients

This multicentre, retrospective analysis was conducted in patients with pediatric malignancies treated with PD-1 inhibitor-based regimens between October 2016 and May 2022 across three academic medical centres in China: Sun Yat-Sen University Cancer Centre, Nanfang Hospital of Southern Medical University, and Fifth Affiliated Hospital of Guangzhou Medical University. The inclusion criteria were as follows:1) pathologically diagnosed with malignancies; 2) age at initiation of PD-1 inhibitor-based therapy <18 years; 3) receiving at least one cycle of PD-1 inhibitor-based regimens; and 4) having measurable lesions. The exclusion criteria were as follows:1) lack of efficacy and toxicity data; and 2) receiving treatment regimens with no more than three patients.

This study was approved by the Institutional Review Board and Ethics Committee of the Sun Yat-sen University Cancer Centre (B2021-064-01) and was conducted in accordance with the Code of Ethics of the World Medical Association (Declaration of Helsinki) for experiments involving humans and Good Clinical Practice. The requirement for written informed consent was waived.

### Data collection and outcome measurement

The medical records of all patients were reviewed. Data regarding demographics, tumour histology, age at initiation of PD-1 inhibitor-based regimens, treatment history, clinical efficacy, and toxicity were retrieved and collected.

PD-1 inhibitor dosing was performed in accordance with the manufacturer’s instructions: nivolumab (3 mg/kg q2w or 240 mg q2w), pembrolizumab (2 mg/kg q3w), toripalimab (3 mg/kg q2w), camrelizumab (200 mg q2w or 200 mg q3w or 3 mg/kg q3w), sintilimab (200 mg q3w), and tislelizumab (200 mg/kg q3w). The doses of all PD-1 inhibitors did not exceed the maximum dose in adults.

The primary objective of this study was to describe the objective response rate (ORR) and progression free survival (PFS) of PD-1 inhibitor-based regimens. ORR is the proportion of patients who achieved complete response (CR) or partial response (PR). PFS was calculated as the date between the initiation of PD-1 inhibitor-based therapy and disease progression or death from any cause. Second, we evaluated the disease control rate (DCR), duration of response (DOR), and treatment-related toxicity. DCR is the proportion of patients who achieved CR, PR, or stable disease (SD). DOR was calculated from the time of the initial response (PR or CR) until death or progression. The Response Evaluation Criteria in Solid Tumours version 1.1 and Lugano classification were used to evaluate the response of solid tumours and lymphoma, respectively. The National Cancer Institute Common Toxicity Criteria for AEs (version 5.0) were used to grade the toxicities.

### Statistical analyses

All statistical analyses were performed using SPSS software (ver. 25, IBM Inc., IL, USA) and R software (ver. 4.2.1; https://www.r-project.org/). A two-sided *P-value <*0.05 was considered significant for all analyses.

## Results

### Study population

**Figure 1:**
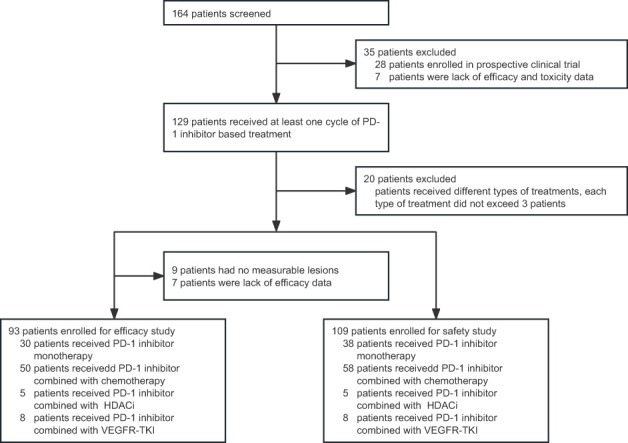
Flow chart of patient screening. PD-1, programmed cell death receptor-1; HDACi, histone deacetylase inhibitor; VEGFR-TKI, vascular endothelial growth factor receptor tyrosine kinase inhibitor.

A total of 109 patients were included in the safety analysis (**Figure 1**). Of those, 93 patients were eligible for efficacy evaluation. The baseline characteristics of all the patients are listed in **Table 1**. The median age at the initiation of PD-1 inhibition-based treatment was 12 years (range 1–18), and the median course of these treatments was 6 (range, 1–27) months. The median follow-up duration was 10.4 (95% CI, 5.5–15.3) months. The majority of patients were males (66.97%). Most patients (95.41%) had good performance status (Karnofsky score 80–100). The tumour histological types of the patients included lymphoma (42/93, 45.16%), bone and soft tissue tumours (19/93, 20.43%), central nervous system tumours (6/93, 6.45%), and other solid tumours (26/93, 27.96%). The detailed pathological types of patients were listed in **Table 1**.

**Table 1:** Baseline characteristics of all patients.

Characteristics	Total (n=109), n (%)
Median age (range) y	12 (1-18)
Median course (range) of PD-1 inhibitor-based treatment	6 (1-27)
Gender	
Female	36 (33.03)
Male	73 (66.97)
Karnofsky score	
≥ 80	104 (95.41)
< 80	5 (4.59)
Histology subgroup (efficacy available)	93 (85.32)
Lymphoma	42 (45.16)
Hodgkin lymphoma	26 (27.96)
Primary mediastinal large B-cell lymphoma	2 (2.15)
EBV-positive diffuse large B-cell lymphoma	2 (2.15)
NK/T-cell lymphoma	11 (11.83)
Subcutaneous Panniculitis Like T-cell Lymphoma	1 (1.08)
Bone and soft tissue tumors	19 (20.43)
Epithelioid sarcoma	3 (3.23)
Mesenchymal malignant tumors	1 (1.08)
Ewing sarcoma	4 (4.30)
Alveolar soft part sarcoma	4 (4.30)
Soft tissue tumor, non-specified	1 (1.08)
Osteosarcoma	2 (2.15)
Embryonal rhabdomyosarcoma	1 (1.08)
Alveolar rhabdomyosarcoma	1 (1.08)
Undifferentiated sarcoma	1 (1.08)
Malignant rhabdoid tumor	1 (1.08)
Central nervous system tumors	6 (6.45)
Anaplastic ependymoma	2 (2.15)
Choroid plexus papilloma	1 (1.08)
Yolk sac tumor of pineal region	1 (1.08)
Glioblastoma	1 (1.08)
Atypical Teratoid/Rhabdoid Tumor	1 (1.08)
Other solid tumors	26 (27.96)
Nasopharyngeal carcinoma	10 (10.75)
Yolk sac tumor	1 (1.08)
Neuroblastoma	3 (3.23)
Hepatoblastoma	2 (2.15)
Malignant pleural mesothelioma	1 (1.08)
Melanoma	2 (2.15)
Lymphoepitheliomatoid carcinoma	4 (4.30)
Renal cell carcinoma	1 (1.08)
Wilms’ tumor	1 (1.08)
Hepatocellular carcinoma	1 (1.08)
Treatment regimen	
PD-1 inhibitor monotherapy	30 (32.26)
PD-1 inhibitor combined with chemotherapy	50 (53.76)
PD-1 inhibitor combined with HDACi	5 (5.38)
PD-1 inhibitor combined with VEGFR-TKI	8 (8.60)
Treatment subgroup	
First-line therapy	25 (26.88)
Salvage therapy	68 (73.12)

PD-1, programmed cell death receptor-1; HDCAi, histone deacetylase inhibitor; VEGFR-TKI, vascular endothelial growth factor receptor tyrosine kinase inhibitor.

The PD-1 inhibitors used in this study included nivolumab and pembrolizumab, which are approved by the United States Food and Drug Administration (FDA), and toripalimab, camrelizumab, sintilimab, and tislelizumab, which are approved by the Chinese FDA (**Supplementary Figure 1**) (12, 17–20). There were no differences in age, gender, karnofsky score, cancer histology, treatment lines, treatment cycles and objective response rate among those treated with six types of PD-1 inhibitors (**Supplementary Table 1**).

The treatment regimens included PD-1 inhibitor monotherapy (n=30), combined chemotherapy (n=50), combined histone deacetylase inhibitor (HDACi) (n=5), and combined vascular endothelial growth factor receptor tyrosine kinase inhibitor (VEGFR-TKI) (n=8) (**Table 1**), with median courses of 7, 4, 10, and 6 courses, respectively (**Supplementary Table 2**). Most patients in the monotherapy cohort (22/30, 73.33%), combined chemotherapy cohort (38/50, 76.00%), and combined HDACi cohort (5/5, 100.00%) have received at least one systematic treatment, while most patients (5/8, 62.50%) in the combined VEGFR–TKI cohort did not receive systematic treatment (**Supplementary Table 2**). Twenty-five (26.88%) and sixty-eight (73.12%) patients received PD-1 based regimens as first-line therapy or salvage therapy, respectively (**Table 1**).

### Antitumor activities for all patients

**Figure 2:**
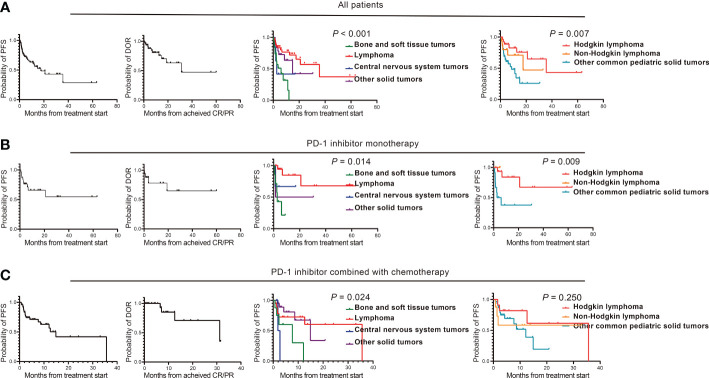
Clinical efficacy of PD-1 inhibitor-based regimens in pediatric malignancies. **(A)** The PFS and DOR of patients who received PD-1 inhibitor-based regimens treatment, the PFS of four types of tumors including bone and soft tissue tumors, lymphoma, central nervous system tumors and other solid tumors, and the PFS of Hodgkin lymphoma, non-Hodgkin lymphoma and other common pediatric solid tumors; **(B)** The PFS and DOR of patients who received PD-1 inhibitor monotherapy, the PFS of four types of tumors including bone and soft tissue tumors, lymphoma, central nervous system tumors and other solid tumors, and the PFS of Hodgkin lymphoma, non-Hodgkin lymphoma and other common pediatric solid tumors; **(C)** The PFS and DOR of patients who received PD-1 inhibitor combined with chemotherapy, the PFS of four types of tumors including bone and soft tissue tumors, lymphoma, central nervous system tumors and other solid tumors, and the PFS of Hodgkin lymphoma, Non-Hodgkin lymphoma and Other common pediatric solid tumors. PD-1, programmed cell death receptor-1; PFS, progression free survival; DOR, duration of response; CR, complete response; PR, partial response.

The efficacy data for the 93 patients are summarised in **Table 2** and **Supplementary Table 3**. Among all the patients, 23 patients (24.73%) achieved CR, 27 patients (29.03%) achieved PR, and the ORR was 53.76%. The patients with CR included Hodgkin lymphoma (n=9), primary mediastinal large B-cell lymphoma (n=1), EBV-positive diffuse large B-cell lymphoma (n=1), extranodal NK/T-cell lymphoma (n=8), anaplastic ependymoma (n=1), nasopharyngeal carcinoma (n=2), and melanoma (n=1). Patients with PR included Hodgkin lymphoma (n=12), primary mediastinal large B-cell lymphoma (n=1), epithelioid sarcoma (n=1), Ewing sarcoma (n=1), nasopharyngeal carcinoma (n=7), hepatoblastoma (n=2), lymphoepithelioma-like carcinoma (n=2), and renal cell carcinoma (n=1). Twenty-six patients (27.96%) achieved SD as best response, and 17 patients (18.28%) developed progressive disease (PD). The DCR was 81.72%. The time of median PFS and median DOR were 17.6 months and 31.2 months, respectively (**Table 2**, **Figure 2A**).

**Table 2:** Antitumor efficacy of PD-1 inhibitor-based regimens.

Clinical evaluation	CR, n (%)	PR, n (%)	SD, n (%)	PD, n (%)	ORR (%)	DCR (%)	Mean PFS (months)	Median PFS (months)	Mean DOR (months)	Median DOR (months)
All patients (n = 93)	23 (24.73)	27 (29.03)	26 (27.96)	17 (18.28)	53.76%	81.72%	27.7 (95% CI 18.4 - 37.0)	17.6 (95% CI 7.9 - 27.2)	36.9 (95% CI 25.4 - 48.3)	31.2
Lymphoma (n = 42)	19 (45.24)	13 (30.95)	6 (14.29)	4 (9.52)	76.19%	90.48%	35.9 (95% CI 23.4 - 48.5)	35.6 (95% CI 9.5 – 61.8)	38.4 (95% CI 25.7 – 51.2)	31.2
Bone and soft tissue tumors (n = 19)	0	2 (10.53)	10 (52.63)	7 (36.84)	10.53%	63.16%	5.9 (95% CI 3.7 - 8.1)	6.0 (95% CI 0.4 – 11.6)	1.5 (95% CI 1.3 – 1.8)	1.3
Central nervous system tumors (n = 6)	1 (16.67)	0	2 (33.33)	3(50.00)	16.67%	50.00%	8.2 (95% CI 1.7 - 14.7)	2.7 (95% CI 1.0 – 4.4)	NA	NA
Other solid tumors (n = 26)	3 (11.54)	12 (46.15)	8 (30.77)	3 (11.54)	57.69%	88.46%	17.6 (95% CI 10.5 - 24.7)	14.9 (95% CI 3.8 – 26.0)	18.9 (95% CI 10.3 – 27.6)	NA
Treatment subgroup										
First-line therapy (n = 25)	3 (12.00)	13 (52.00)	6 (24.00)	3 (12.00)	64.00%	88.00%	23.2 (95% CI 17.1 - 29.2)	NA	26.8 (95% CI 17.1 - 29.2)	30.8
Salvage therapy (n = 68)	20 (29.41)	14 (20.59)	20 (29.41)	14 (20.59)	50.00%	79.41%	24.7 (95% CI 15.6 - 33.9)	14.9 (95% CI 5.6 - 24.2)	34.1 (95% CI 22.2 - 46.1)	31.2 (95% CI 5.5 - 56.9)
PD-1 inhibitor monotherapy (n = 30)	7 (23.33)	10 (33.33)	8 (26.67)	5 (16.67)	56.67%	83.33%	38.0 (95% CI 25.1 - 51.0)	NA	42.0 (95% CI 27.0 - 57.0)	NA
Lymphoma (n = 18)	5 (27.78)	9 (50.00)	4 (22.22)	0	77.78%	100.00%	47.2 (95% CI 31.2 - 63.2)	NA	41.8 (95% CI 24.4 - 59.1)	NA
Bone and soft tissue tumors (n = 7)	0	1(14.29)	3(42.86)	3(42.86)	14.29%	57.14%	4.2 (95% CI 1.9 - 6.5)	2.7 (95% CI 2.0 - 3.5)	1.3 (95% CI 1.3 – 1.3)	1.3
Central nervous system tumors (n = 3)	1 (33.33)	0	1(33.33)	1(33.33)	33.33%	66.67%	11.7 (95% CI 3.3 - 20.1)	NA	NA	NA
Other solid tumors (n = 2)	1 (50.00)	0	0	1(50.00)	50.00%	50.00%	16.0 (95% CI 0 - 36.1)	1.4	NA	NA
PD-1 inhibitor combined with chemotherapy (n = 50)	11 (22.00)	16 (32.00)	13 (26.00)	10 (20.00)	54.00%	80.00%	19.0 (95% CI 13.0 - 25.0)	14.9 (95% CI 10.6 - 19.2)	25.1 (95% CI 18.8 - 31.4)	31.2 (95% CI 4.2 - 58.2)
Lymphoma (n = 19)	9 (47.37)	4(21.05)	2(10.53)	4(21.05)	68.42%	78.95%	23.5 (95% CI 14.9 - 32.1)	35.6	28.4 (95% CI 22.8 – 33.9)	31.2 (95% CI 0.0 – 65.2)
Bone and soft tissue tumors (n = 8)	0	1(12.50)	4(50.00)	3(37.50)	12.50%	62.50%	6.5 (95% CI 2.6 - 10.5)	7.7 (95% CI 0 - 15.7)	NA	NA
Central nervous system tumors (n = 3)	0	0	1(33.33)	2(66.67)	0	33.33%	2.3 (95% CI 1.5 - 3.1)	1.9	NA	NA
Other solid tumors (n = 20)	2(10.00)	11(55.00)	6(30.00)	1(5.00)	65.00%	95.00%	13.7 (95% CI 9.3 - 18.2)	14.9 (95% CI 5.8 - 24.0)	14.0 (95% CI 9.1 – 18.9)	12.6 (95% CI 3.8 – 21.3)
PD-1 inhibitor combined with HDACi (n = 5)	5 (100.00)	0	0	0	100.00%	100.00%	15.2 (95% CI 11.0 - 19.4)	17.6 (95% CI 0.2 - 34.9)	12.9 (95% CI 8.7 - 17.1)	14.9 (95% CI 0 - 31.7)
NK/T-cell lymphoma (n = 5)	5(100.00)	0	0	0	100.00%	100.00%	15.2 (95% CI 11.0 - 19.4)	17.6 (95% CI 0.2 - 34.9)	12.9 (95% CI 8.7 - 17.1)	14.9 (95% CI 0 - 31.7)
PD-1 inhibitor combined with VEGFR-TKI (n = 8)	0	1 (12.50)	5 (62.50)	2 (25.00)	12.50%	75.00%	6.8 (95% CI 3.3 - 10.3)	3.7 (95% CI 0 - 7.9)	1.8 (95% CI 1.8 – 1.8)	1.8
Bone and soft tissue tumors (n = 4)	0	0	3(75.00)	1(25.00)	0	75.00%	6.6 (95% CI 1.0 - 12.3)	3.7 (95% CI 0.0 - 10.8)	NA	NA
Other solid tumors (n = 4)	0	1(25.00)	2(50.00)	1(25.00)	25.00%	75.00%	6.6 (95% CI 2.8 - 10.4)	3.6	NA	NA

PD-1, programmed cell death receptor-1; HDACi, histone deacetylase inhibitor; VEGFR-TKI, vascular endothelial growth factor receptor tyrosine kinase inhibitor; CR, complete response; PR, partial response; SD, stable disease; PD, progressive disease; ORR, objective response; DCR, disease control rate; PFS, progression free survival; DOR, duration of response; NA, not achieved.

Among the patients with different tumour histological types, the ORR were 76.19%, 10.53%, 16.67%, and 57.69% for lymphoma, bone and soft tissue tumours, central nervous system tumours, and other solid tumours, respectively. The PFS between the four-types of malignancies was significantly statistically different (*P <*0.001); the median PFS duration of lymphoma, bone and soft tissue tumours, central nervous system tumours, and other solid tumours were 35.6, 6.0, 2.7, and 14.9 months (95% CI, 3.8–26.0), respectively (**Figure 2A**). The median DOR were 31.2 months, 1.3 months, not achieved (NA), and NA for lymphoma, bone and soft tissue tumours, central nervous system tumours, and other solid tumours (**Table 2**).

The ORR and DCR of Hodgkin lymphoma, non-Hodgkin lymphoma, and other common pediatric solid tumours were 80.77%/96.15%, 68.75%/81.25%, and 35.29%/74.51%, respectively. Their median PFS durations were significantly different (*P*=0.007): 35.6, 17.6, and 8.6 months, respectively (**Figure 2A**). The median DOR was 31.2 months, NA, and NA for Hodgkin lymphoma, non-Hodgkin lymphoma, and other common pediatric solid tumours (**Supplementary Table 3**).

Among the patients who received PD-1 inhibitor-based regimen as first-line therapy, there were 3 patients (12.00%) with CR and 13 patients (52.00%) with PR, and the ORR was 64.00%. Six patients (24.00%) achieved SD as best response and three patients (12.00%) developed PD, with 88% DCR. For patients who received PD-1 inhibitor-based regimens as salvage therapy, 20 patients (29.41%) achieved CR and 14 patients (20.59%) achieved PR, and the ORR was 50.00%. Twenty patients (29.41%) achieved SD as best response and 14 patients (20.59%) developed PD. The DCR was 79.41% (**Table 2**).

### Antitumor activities for PD-1 inhibitor monotherapy

In the monotherapy cohort, the ORR was 56.67%, including seven patients (23.33%) with CR and 10 patients (33.33%) with PR. Hodgkin’s lymphoma (n=4), extranodal NK/T-cell lymphoma (n=1), anaplastic ependymoma (n=1), and melanoma (n=1) achieved CR as the best response. Hodgkin lymphoma (n=8), primary mediastinal large B-cell lymphoma (n=1), and Ewing sarcoma (n=1) showed PR as the best response. Eight patients (26.67%) achieved SD as best response, and five patients (16.67%) developed PD. The DCR was 83.33%. Both the median PFS and DOR were NA (**Table 2**, **Figure 2B**).

The ORR were 77.78%, 14.29%, 33.33%, and 50.00% for lymphoma, bone and soft tissue tumours, central nervous system tumours, and other solid tumours, respectively (**Table 2**). The median PFS durations were NA, 2.7 months, NA, and 1.4 months for lymphoma, bone and soft tissue tumours, central nervous system tumours, and other solid tumours, respectively, with significantly statistically different (*P*=0.014) (**Figure 2B**).

The ORR and DCR were 80.00%/100.00%, 66.67%/100.00%, and 25.00%/58.33% for Hodgkin lymphoma, non-Hodgkin lymphoma, and other common pediatric solid tumours, respectively, and their PFS durations were statistically different (NA, NA, and 2.7 months; **Figure 2B**). The median DOR was NA (**Supplementary Table 3**).

### Antitumor activities for PD-1 inhibitor combined with chemotherapy

In the combined chemotherapy cohort, 11 patients (22.00%) achieved CR, 16 patients (32.00%) achieved PR, and the ORR was 54.00%. The tumour types for the patients with CR included Hodgkin’s lymphoma (n=5), primary mediastinal large B-cell lymphoma (n=1), EBV-positive diffuse large B-cell lymphoma (n=1), extranodal NK/T-cell lymphoma (n=2), and nasopharyngeal carcinoma (n=2). The tumour types for the patients with PR included Hodgkin’s lymphoma (n=4), epithelioid sarcoma (n=1), nasopharyngeal carcinoma (n=7), lymphoepithelioma-like carcinoma (n=2), and hepatoblastoma (n=2). Thirteen patients (26.00%) achieved SD as best response and 10 patients (20.00%) developed PD. The DCR was 80.00%. The PFS median duration and median DOR were 14.9 and 31.2 months, respectively (**Table 2**, **Figure 2C**).

The ORR for lymphoma, bone and soft tissue tumours, central nervous system tumours, and other solid tumours were 68.42%, 12.50%, 0, and 65.00%, respectively. The PFS between the four-types of malignancies was significantly statistically different (*P*=0.024): the median PFS duration of lymphoma, bone and soft tissue tumours, central nervous system tumours, and other solid tumours were 35.6, 7.7, 1.9, and 14.9 months, respectively (**Table 2**, **Figure 2C**).

The ORR and DCR of Hodgkin lymphoma, non-Hodgkin lymphoma, and other common pediatric solid tumours were 81.82%/90.91%, 50.00%/62.50%, and 45.16%/80.65%, respectively, and their median PFS durations were 35.6 months, NA, and 12.1 months, respectively (**Figure 2C**). The median DOR were 31.2 months, NA and 12.6 months for Hodgkin lymphoma, non-Hodgkin lymphoma, and other common pediatric solid tumours (**Supplementary Table 3**).

### Antitumor activities for PD-1 inhibitor combined with HDACi or VEGFR-TKI

In the combined HDACi cohort, all 5 patients with extranodal NK/T-cell lymphoma achieved CR. The ORR and DCR were 100.00%. The median PFS duration and median DOR were 17.6 and 14.9 months, respectively (**Table 2**, **Supplementary Figures 2A, B**). Among the patients in the combined VEGFR-TKI cohort, there were no patients with CR and one renal cell carcinoma patient (12.50%) with PR, and the ORR was 12.50%. Five patients (62.50%) achieved SD as best response and two patients (25.00%) developed PD. The DCR was 75.00%. The median PFS duration and DOR were 3.7 and 1.8 months, respectively (**Table 3**, **Supplementary Figure 2C**).

**Table 3:** Profile of adverse events of PD-1 inhibitor-based treatment.

Adverse events	All patients (n = 109, %)	PD-1 inhibitor monotherapy (n = 38, %)	PD-1 inhibitor combined with chemotherapy (n = 58, %)	PD-1 inhibitor combined with HDACi (n = 5, %)	PD-1 inhibitor combined with VEGFR-TKI (n = 8, %)
Overall adverse events	91 (83.49)	21 (55.26)	58 (100.00)	4 (80.00)	8 (100.00)
Grade 1-2 adverse events	91 (83.49)	21 (55.26)	58 (100.00)	4 (80.00)	8 (100.00)
Grade 3-4 adverse events	40 (36.70)	0	37 (63.79)	2 (40.00)	1 (12.50)
Hematological adverse events					
Anemia	46 (42.20)	2 (5.26)	41 (70.69)	2 (40.00)	1 (12.50)
Platelet count decreased	30 (27.52)	2 (5.26)	26 (44.83)	0	2 (25.00)
White blood cell decreased	59 (54.13)	5 (13.16)	46 (79.31)	4 (80.00)	4 (50.00)
Constitutional adverse events					
Fatigue	21 (19.27)	4 (10.53)	15 (25.86)	1 (20.00)	1 (12.50)
Fever	18 (16.51)	6 (15.79)	9 (15.52)	2 (40.00)	1 (12.50)
Weight loss	6 (5.50)	0	5 (8.62)	1 (20.00)	0
Hypersomnia	9 (8.26)	0	7 (12.07)	1 (20.00)	1 (12.50)
Skin and mucosa adverse events					
Skin hyperpigmentation	14 (12.84)	0	14 (24.14)	0	0
Rash	16 (14.68)	3 (7.89)	8 (13.79)	0	5 (62.50)
Alopecia	22 (20.18)	1 (2.63)	18 (31.03)	1 (20.00)	2 (25.00)
Mucositis oral	11 (10.09)	0	9 (15.52)	1 (20.00)	1 (12.50)
Respiratory adverse events					
Lung infection	1 (0.92)	0	1 (1.72)	0	0
Gastrointestinal adverse events					
Anorexia	23 (21.10)	3 (7.89)	19 (32.76)	0	1 (12.50)
Nausea	46 (42.20)	2 (5.26)	39 (67.24)	2 (40.00)	3 (37.50)
Vomiting	33 (30.28)	2 (5.26)	27 (46.55)	1 (20.00)	3 (37.50)
Abdominal pain	11 (10.09)	1 (2.63)	7 (12.07)	1 (20.00)	2 (25.00)
Diarrhea	10 (9.17)	1 (2.63)	5 (8.62)	1 (20.00)	3 (37.50)
Aspartate aminotransferase increased	15 (13.76)	1 (2.63)	11 (18.97)	1 (20.00)	2 (25.00)
Alanine aminotransferase increased	12 (11.01)	1 (2.63)	10 (17.24)	1 (20.00)	0
Musculoskeletal adverse events					
Myalgia	3 (2.75)	0	2 (3.45)	0	1 (12.50)
Endocrine adverse events					
Hypothyroidism	16 (14.68)	4 (10.53)	7 (12.07)	2 (40.00)	3 (37.50)
Diabetic ketoacidosis	1 (0.92)	0	1 (1.72)	0	0

PD-1, programmed cell death receptor-1; HDACi, histone deacetylase inhibitor; VEGFR-TKI, vascular endothelial growth factor receptor tyrosine kinase inhibitor.

### Safety and toxicity

The safety data of 109 patients are listed in **Table 3**. We only presented the AEs occurring in ≥10% of patients. AEs occurring in <10% of the population are shown when there was a corresponding event in grades 3–5. In all the patients, the incidence rates of all grade and grade 3 or higher AEs were 83.49% (91/109) and 36.70% (40/109), respectively. The overall incidence rates of AEs in PD-1 inhibitor monotherapy, combined chemotherapy, combined HDACi, and combined VEGFR-TKI cohorts were 55.26% (21/38), 100.00% (58/58), 80.00% (4/5), and 100.00% (8/8), respectively. The most common AEs were fever, decrease in the white blood cell (WBC) count, decrease in the WBC count, and rash, respectively. All AEs in the monotherapy cohort were grade 1–2. Thirty-seven patients (63.79%), two patients (40.00%), and one patient (12.50%) developed AEs of grade 3 or higher in combined chemotherapy, combined HDACi, and combined VEGFR-TKI cohorts, respectively. The most common grade 3–4 adverse event of combined chemotherapy was the decrease in the platelet count (34.48%, 20/58). Grade 3–4 AEs of combined HDACi included increase in aspartate aminotransferase (20.00%, 1/5) and decrease in WBC count (20.00%, 1/5). Myalgia (12.50%, 1/8) was the only grade 3–4 AE in combined VEGFR-TKI cohort. No fatal toxicity was observed in any patient.

Potential immune-related AEs including myositis, hypothyroidism, and diabetic ketoacidosis were observed. One patient in the combined VEGFR-TKI cohort developed immune-related myositis (grade 2) and recovered with prednisone treatment. All hypothyroidism cases were grade 1–2. The incidence of hypothyroidism in monotherapy, combined chemotherapy, combined HDACi, and combined VEGFR TKI cohorts were 10.53% (4/38), 12.07% (7/58), 40.00% (2/5), and 37.50% (3/8), respectively. In the combined chemotherapy cohort, one patient developed diabetic ketoacidosis and discontinued PD-1 inhibitor treatment.

## Discussion

The ORR of Hodgkin lymphoma, non-Hodgkin lymphoma, and non-lymphoma were 22.22%-60.00%, 0-33.33%, 0-5.97%, respectively, as reported in phase 1–2 clinical studies that assessed the clinical activity of nivolumab, pembrolizumab, and atezolizumab monotherapy in pediatric malignancies (11–13). The present study showed higher ORR in Hodgkin lymphoma (80.00%), non-Hodgkin lymphoma (66.67%), and other common pediatric solid tumours (25.00%) in the cohort receiving PD-1 inhibitor monotherapy. This could be attributed to the advanced timing of PD-1 inhibitor administration. The median value of previous lines of systematic therapy in this study was 1 (range, 0–6) compared to 2.5, 2, and 6 in the previous studies(11–13); Moreover, the non-Hodgkin lymphoma in our study included primary mediastinal large B-cell lymphoma and extranodal NK/T-cell lymphoma which have a better response to PD-1 inhibitor treatment (21–23).

We reported the efficacy of PD-1 inhibitors combined with chemotherapy in nasopharyngeal carcinoma and lymphoepithelioma-like carcinoma in children, while previous studies mainly focused on adults (24–26). The ORR for ten patients with nasopharyngeal receiving combined chemotherapy was 90% (CR: 2, PR: 7, SD: 1), and the ORR for four patients with lymphoepithelioma-like carcinoma was 50% (PR:2, SD:2). A previous study reported that hepatoblastoma was not responsive to PD-1 inhibitor monotherapy (12). Two patients with refractory/relapsed hepatoblastoma receiving PD-1 inhibitor combined with chemotherapy achieved PR as best response in present study. Our findings suggest that PD-1 inhibitors combined with chemotherapy could be a good therapeutic option for pediatric patients with these tumours.

Previous studies have revealed that HDACi can enhance the activity of PD-1 inhibitors in refractory/relapsed extranodal NK/T-cell lymphoma (22, 27). There is an ongoing clinical study exploring the efficacy of PD-1 inhibitors in combination with chidamide (NCT03820596). Consistently, five patients who received a PD-1 inhibitor combined with HDACi achieved CR in this study, which further verifies the efficacy of this regimen in extranodal NK/T-cell lymphoma. The combination of PD-1 inhibitors and VEGFR-TKIs shows promising efficacy and safety in adult tumours (28, 29). Anti-angiogenic agents can stabilise the vessels of the tumour and reverse the immunosuppressive microenvironment to synergistically improve the anti-tumour effect of PD-1 inhibitors (30, 31). There are ongoing clinical studies on PD-1 inhibitors combined with VEGFR-TKIs. The ORR of this combined therapy was relatively low (12.50%, 1/8) in our study, but the sample size was too small to appropriately reflect the efficacy. A prospective large-cohort study is needed to provide more reliable results.

Studies involving PD-1 inhibitor monotherapy in both adults and children have shown a low incidence of AEs with good tolerability and rare incidence of severe events. The overall incidence rate of AEs of PD-1 inhibitor monotherapy in adult patients was 69.7%–70% (32–34). Pembrolizumab was reported to be safe and tolerable in pediatric patients, where around 56% of the patients experienced all grade-AEs and 8% of the patients experienced grade 3–5 events (12). In our study, AEs occurred in 52.63% of the patients, but and no grade 3–4 events were observed, indicating a better safety profile of PD-1 inhibitors in children than adults.

Owing to the small sample size in the combined HDACi and VEGFR-TKI cohorts, we mainly focused on the combined chemotherapy cohort. Our results revealed that the incidence rates of all-grade and grade 3–4 adverse event were 100% and 63.79% in the pediatric patients receiving PD-1 inhibitor combined with chemotherapy, which were comparable to the data in the adult patients (35, 36). In addition, the profile and incidence rate of all-grade and grade 3–4 AEs in pediatric patients in our study were consistent with those of chemotherapy in other studies (37–39). These findings suggest that PD-1 combined with chemotherapy is safe, and that AEs are associated with chemotherapy in pediatric patients. One patient developed type 1 diabetes presenting with diabetic ketoacidosis after three cycles of treatment with PD-1 inhibitor combined with chemotherapy and required permanent exogenous insulin treatment. The prevalence of immune-related type 1 diabetes is approximately 1% in adult patients receiving immune checkpoint-inhibitor treatment and the window for disease development is within the first three months of starting therapy (40). In previous studies on pediatric patients, only one patient developed diabetic ketoacidosis from atezolizumab therapy (13). The occurrence of immune-related type 1 diabetes might be due to an autoreactive CD8^+^ T cell clone which was activated after PD-1 inhibitor therapy. Insulin-producing pancreatic beta cells cannot evade the damage through binding to the PD-1 receptor on autoreactive CD8^+^ T cells (41).

The PD-1 inhibitors approved by the United States FDA are relatively more expensive and less accessible than those developed in China. A phase I clinical study is being conducted by our institution to explore the efficacy and safety of the PD-1 antibody, sintilimab, in patients with refractory or relapsed pediatric cancer (NCT04400851). Our study has some limitations due to its retrospective design. First, the selective bias was almost inevitable. Second, there was a limited sample size for multiple types of tumours. Third, there were multiple types of PD-1 inhibitors. Despite these limitations, this study revealed that PD-1 inhibitor-based therapy has promising efficacy and tolerable AEs in pediatric malignancies, especially lymphoma.

In conclusion, we demonstrated that PD-1 inhibitor-based treatment shows promising efficacy and tolerable AEs in a large real-world cohort of pediatric malignancies in China. Our findings provide evidence of the clinical trial and practice of PD-1 inhibitor-based therapies in pediatric patients. Further prospective studies are required to determine the optimal treatment strategies for pediatric patients.

## Data availability statement

The raw data supporting the conclusions of this article will be made available by the authors, without undue reservation.

## Ethics statement

The retrospective study was approved by the institutional review board and ethics committee of the Sun Yat-Sen University Cancer Center with approval number B2021-064-01. Given the retrospective design of the study and the anonymous nature of the data, the ethical committee waived the requirement of informed consent.

## Author contributions

YZZ and YQ designed the research. YH, MS, YL, JW, SL, YZ, JZ, FS, JH, JL, JX, YW, HG, RC and ZZ collected and analysed data. YH, MS and YL wrote and revised the manuscript. All authors contributed to the article and approved the submitted version.
