# Supplementary material for: Efficacy and safety of programmed cell death receptor 1 inhibition-based regimens in patients with pediatric malignancies: the real-world study in China
Source: Front Immunol. 2023 Jun 9;14:1182751. doi: 10.3389/fimmu.2023.1182751 (PMC10288191; doi:10.3389/fimmu.2023.1182751)
Supplement: Supplementary file 1 [file DataSheet_1.docx]

Supplementary Material

**Efficacy and Safety of Programmed Cell Death Receptor 1 Inhibition-Based Regimens in Patients with Pediatric Malignancies: The Real-World Study in China**

**Ye Hong, Mengjia Song, Yingxia Lan, Juan Wang, Suying Lu, Yu Zhang, Jia Zhu, Feifei Sun, Junting Huang, Juan Liu, Jiaqian Xu, Yanpeng Wu, Haixia Guo, Ruiqing Cai, Zijun Zhen, Yi Que, Yizhuo Zhang**

*** Correspondence:** Prof. Yi Z. Zhang, Department of Pediatric Oncology, Sun Yat-sen University Cancer Center. 651 Dongfeng East Road, Guangzhou, Guangdong 510060, P. R. China. Tel: +86-20-87342466; Fax: +86-20-87343598; Email: zhangyzh@sysucc.org.cn; Dr. Yi Que, Department of Pediatric Oncology, Sun Yat-sen University Cancer Center. 651 Dongfeng East Road, Guangzhou, Guangdong 510060, P. R. China. Tel: +86-20-87342466; Fax: +86-20-87343598; Email: [queyi@sysucc.org.cn](mailto:queyi@sysucc.org.cn).

# Supplementary Figures and Tables

## Supplementary Figures


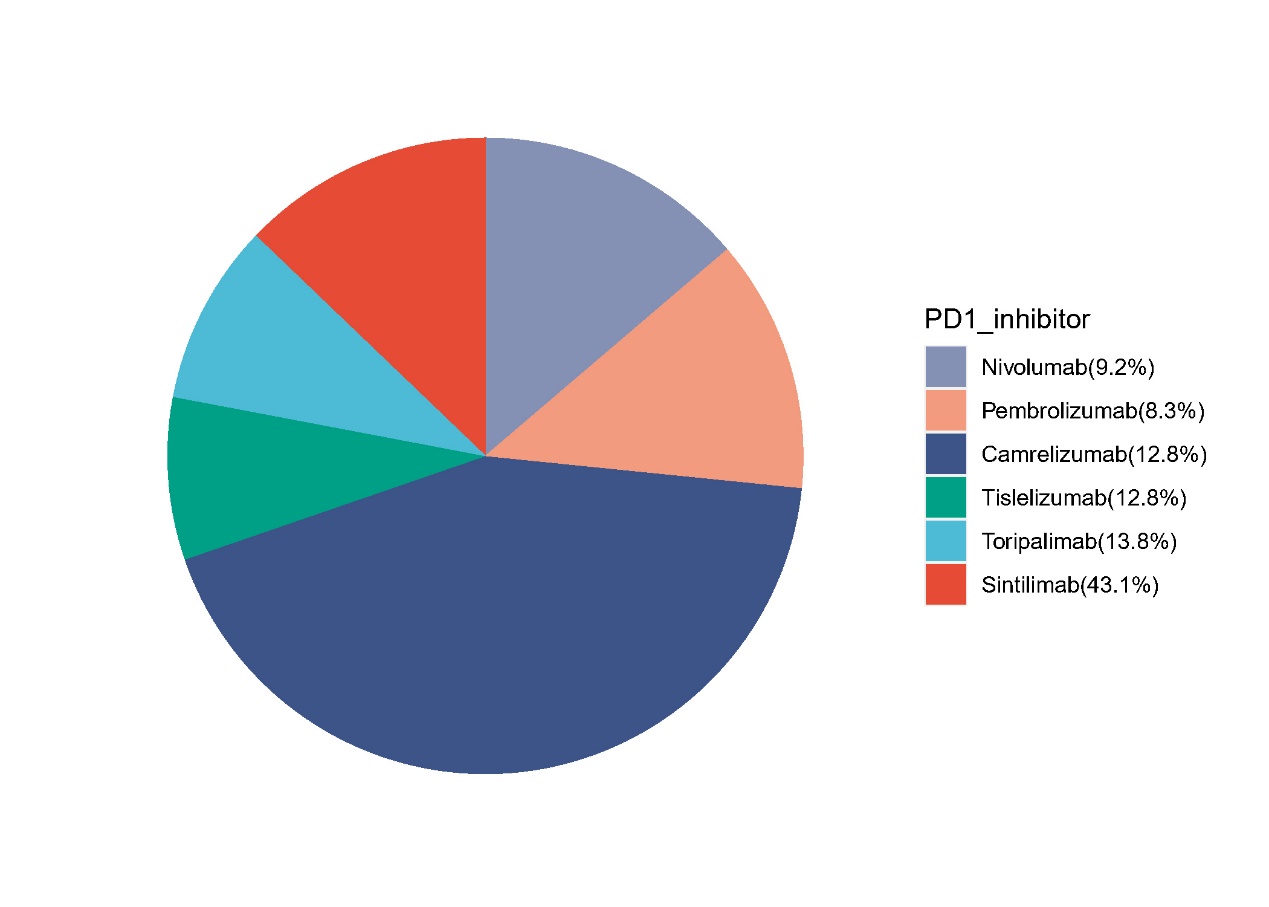


**Supplementary Figure 1.** The proportion of six-kind of PD-1 inhibitor used in this study.


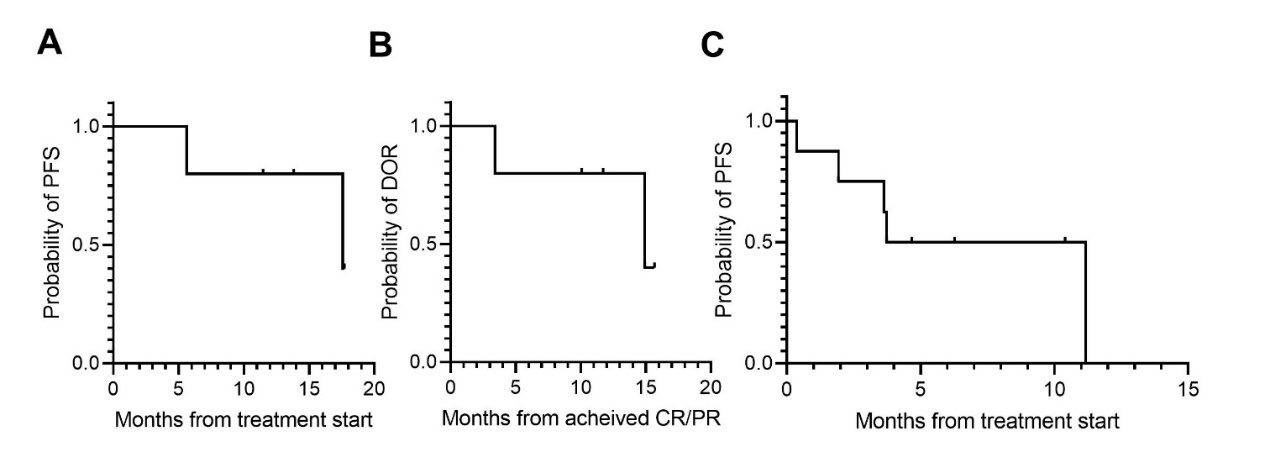


**Supplementary Figure 2.** Clinical efficacy of PD-1 inhibitor-based regimens in pediatric malignancies. (A, B) The PFS and DOR of patients who received PD-1 inhibitor combined with HDACi; (C) The PFS of patients who received PD-1 inhibitor combined with VEGFR-TKI. Abbreviations: PD-1, programmed cell death receptor 1; PFS, progression free survival; DOR, duration of response; CR, complete response; PR, partial response; HDACi, histone deacetylase inhibitor; VEGFR-TKI, vascular endothelial growth factor receptor tyrosine kinase inhibitor.

## Supplementary Tables

**Supplementary Table 1. Baseline characteristics of patients received six types of PD-1 inhibitors.**

| **Characteristics** | No. of efficacy evaluable patients (%) | | | | | | | *P* value | |  |
| --- | --- | --- | --- | --- | --- | --- | --- | --- | --- | --- |
|  | All patients  (n =93) | Nivolumab  (n = 8) | Pembrolizumab  (n = 7) | Camrelizumab  (n = 9) | Tislelizumab  (n = 13) | Toripalimab  (n = 13) | Sintilimab  (n = 43) |  |  |  |
| **Median age (range), years** | 12 (1-18) | 9 (1-17) | 11 (3-15) | 13 (6-16) | 14 (6-16) | 13 (4-17) | 11 (3-18) | 0.286 |  | |
| **Gender** |  |  |  |  |  |  |  |  |  | |
| Male | 63 (67.74) | 6 (75.00) | 6 (85.71) | 7 (77.78) | 9 (69.23) | 9 (69.23) | 26 (60.47) | 0.823 |  | |
| Female | 30 (32.26) | 2 (25.00) | 1 (14.29) | 2 (22.22) | 4 (30.77) | 4 (30.77) | 17 (39.53) |  |  | |
| **Karnofsky score** |  |  |  |  |  |  |  |  |  | |
| ≥ 80 | 89 (95.70) | 7 (87.50) | 7 (100.00) | 9 (100.00) | 13 (100.00) | 13 (100.00) | 40 (93.02) | 0.685 |  | |
| < 80 | 4 (4.30) | 1 (12.50) | 0 | 0 | 0 | 0 | 3 (6.98) |  |  | |
| **Histology** |  |  |  |  |  |  |  | 0.061 |  | |
| Hodgkin lymphoma | 26 (27.96) | 5 (62.50) | 0 | 1 (11.11) | 6 (46.15) | 2 (15.38) | 12 (27.91) |  |  | |
| Primary mediastinal large B-cell lymphoma | 2 (2.15) | 0 | 1 (14.29) | 0 | 0 | 0 | 1 (2.33) |  |  | |
| EBV-positive diffuse large B-cell lymphoma | 2 (2.15) | 0 | 0 | 0 | 0 | 1 (7.69) | 1 (2.33) |  |  | |
| NK/T-cell lymphoma | 11 (11.83) | 0 | 1 (14.29) | 2 (22.22) | 1 (7.69) | 2 (15.38) | 5 (11.63) |  |  | |
| Subcutaneous Panniculitis Like T-cell Lymphoma | 1 (1.08) | 0 | 0 | 0 | 0 | 1 (7.69) | 0 |  |  | |
| Epithelioid sarcoma | 3 (3.23) | 0 | 0 | 0 | 0 | 0 | 3 (6.98) |  |  | |
| Mesenchymal malignant tumors | 1 (1.08) | 0 | 0 | 0 | 0 | 0 | 1 (2.33) |  |  | |
| Ewing sarcoma | 4 (4.30) | 0 | 1 (14.29) | 0 | 0 | 1 (7.69) | 2 (4.65) |  |  | |
| Alveolar soft part sarcoma | 4 (4.30) | 0 | 1 (14.29) | 3 (33.33) | 0 | 0 | 0 |  |  | |
| Soft tissue tumor, non-specified | 1 (1.08) | 0 | 0 | 0 | 1 (7.69) | 0 | 0 |  |  | |
| Osteosarcoma | 2 (2.15) | 0 | 0 | 2 (22.22) | 0 | 0 | 0 |  |  | |
| Embryonal rhabdomyosarcoma | 1 (1.08) | 0 | 0 | 0 | 0 | 0 | 1 (2.33) |  |  | |
| Alveolar rhabdomyosarcoma | 1 (1.08) | 0 | 1 (14.29) | 0 | 0 | 0 | 0 |  |  | |
| Undifferentiated sarcoma | 1 (1.08) | 0 | 0 | 0 | 0 | 0 | 1 (2.33) |  |  | |
| Malignant rhabdoid tumor | 1 (1.08) | 0 | 0 | 0 | 0 | 1 (7.69) | 0 |  |  | |
| Anaplastic ependymoma | 2 (2.15) | 0 | 0 | 0 | 0 | 0 | 2 (4.65) |  |  | |
| Choroid plexus papilloma | 1 (1.08) | 0 | 0 | 0 | 0 | 0 | 1 (2.33) |  |  | |
| Yolk sac tumor of pineal region | 1 (1.08) | 0 | 0 | 0 | 0 | 0 | 1 (2.33) |  |  | |
| Glioblastoma | 1 (1.08) | 1 (12.50) | 0 | 0 | 0 | 0 | 0 |  |  | |
| Atypical Teratoid/Rhabdoid Tumor | 1 (1.08) | 0 | 0 | 0 | 0 | 1 (7.69) | 0 |  |  | |
| Nasopharyngeal carcinoma | 10 (10.75) | 0 | 0 | 1 (11.11) | 3 (23.08) | 4 (30.77) | 2 (4.65) |  |  | |
| Yolk sac tumor | 1 (1.08) | 0 | 0 | 0 | 0 | 0 | 1 (2.33) |  |  | |
| Neuroblastoma | 3 (3.23) | 2 (25.00) | 0 | 0 |  | 0 | 1 (2.33) |  |  | |
| Hepatoblastoma | 2 (2.15) | 0 | 1 (14.29) | 0 | 1 (7.69) | 0 | 0 |  |  | |
| Malignant pleural mesothelioma | 1 (1.08) | 0 | 0 | 0 | 0 | 0 | 1 (2.33) |  |  | |
| Melanoma | 2 (2.15) | 0 | 1 (14.29) | 0 | 0 | 0 | 1 (2.33) |  |  | |
| Lymphoepitheliomatoid carcinoma | 4 (4.30) | 0 | 0 | 0 | 0 | 0 | 4 (9.30) |  |  | |
| Renal cell carcinoma | 1 (1.08) | 0 | 0 | 0 |  | 0 | 1 (2.33) |  |  | |
| Wilms’ tumor | 1 (1.08) | 0 | 0 | 0 | 0 | 0 | 1 (2.33) |  |  | |
| Hepatocellular carcinoma | 1 (1.08) | 0 | 0 | 0 | 1 (7.69) | 0 | 0 |  |  | |
| **Treatment lines** |  |  |  |  |  |  |  | 0.136 |  | |
| 1 | 25 (26.88) | 0 | 2 (28.57) | 3 (33.33) | 7 (53.85) | 3 (23.08) | 10 (23.26) |  |  | |
| ≥ 2 | 68 (73.12) | 8 (100.00) | 5 (71.43) | 6 (66.67) | 6 (46.15) | 10 (76.92) | 33 (76.74) |  |  | |
| **Treatment regimens** |  |  |  |  |  |  |  | 0.728 |  | |
| Monotherapy | 30 (32.26) | 3 (37.50) | 2 (28.57) | 1 (11.11) | 5 (38.46) | 2 (15.38) | 17 (39.53) |  |  | |
| Combined with chemotherapy | 50 (53.76) | 5 (62.50) | 4 (57.14) | 5 (55.56) | 6 (46.15) | 10 (76.92) | 20 (46.51) |  |  | |
| Combined with HDACi | 5 (5.38) | 0 | 0 | 1 (11.11) | 1 (7.69) | 0 | 3 (6.98) |  |  | |
| Combined with VEGFR-TKI | 8 (8.60) | 0 | 1 (14.29) | 2 (22.22) | 1 (7.69) | 1 (7.69) | 3 (6.98) |  |  | |
| **Cycles of treatment, median (range), No** | 6 (1-27) | 7 (2-16) | 9 (1-15) | 6 (3-19) | 5 (2-21) | 6 (2-27) | 6 (1-21) | 0.886 |  | |
| **Objective response rate** | 50/93 (53.76) | 4/8 (50.00) | 4/7 (57.14) | 3/9 (33.33) | 9/13 (69.23) | 8/13 (61.54) | 22/43 (51.16) | 0.6761 |  | |

Abbreviations: PD-1, programmed cell death receptor 1; HDCAi, histone deacetylase inhibitor; VEGFR-TKI, vascular endothelial growth factor receptor tyrosine kinase inhibitor.

# Supplementary Table 2. Baseline characteristics of four-cohort of patients receiving PD-1 inhibitor-based treatment

| **Characteristics** | **PD-1 inhibitor monotherapy (n = 30)** | **PD-1 inhibitor combined with chemotherapy (n = 50)** | **PD-1 inhibitor combined with HDACi (n = 5)** | **PD-1 inhibitor combined with VEGFR-TKI (n = 8)** |
| --- | --- | --- | --- | --- |
| **Median age (range) y** | 11 (3 - 18) | 13 (1 - 17) | 12 (6 - 17) | 8 (3 - 16) |
| **Median course (range) of PD-1 inhibitor-based treatment** | 7 (1 - 27) | 4 (1 - 17) | 10 (7 - 19) | 6 (1 - 18) |
| **Gender** |  |  |  |  |
| Female | 12 (40.00) | 13 (26.00) | 2 (40.00) | 3 (37.50) |
| Male | 18(60.00) | 37 (74.00) | 3 (60.00) | 5 (62.50) |
| **Karnofsky score** |  |  |  |  |
| ≥ 80 | 30 (100.00) | 46 (92.00) | 5 (100.00) | 8 (100.00) |
| < 80 | 0 | 4 (8.00) | 0 | 0 |
| **Previous systematic treatment lines** |  |  |  |  |
| 0 | 8 (26.67) | 12 (24.00) | 0 | 5 (62.50) |
| ≥ 1 | 22 (73.33) | 38 (76.00) | 5 (100.00) | 3 (37.50) |

Abbreviations: PD-1, programmed cell death receptor 1; HDCAi, histone deacetylase inhibitor; VEGFR-TKI, vascular endothelial growth factor receptor tyrosine kinase inhibitor.

**Supplementary Table 3. Clinical efficacy of PD-1 inhibitor-based treatment in lymphoma and other pediatric malignancies.**

| **Clinical evaluation** | **All patients n (%)** | **PD-1 inhibitor monotherapy n (%)** | **PD-1 inhibitor combined with chemotherapy n (%)** | **PD-1 inhibitor combined with HDACi n (%)** | **PD-1 inhibitor combined with VEGFR-TKI n (%)** |
| --- | --- | --- | --- | --- | --- |
| **ORR** |  |  |  |  |  |
| Hodgkin lymphoma | 21/26 (80.77) | 12/15 (80.00) | 9/11 (81.82) | 0 | 0 |
| Non-Hodgkin lymphoma | 11/16 (68.75) | 2/3(66.67) | 4/8 (50.00) | 5/5 (100.00) | 0 |
| Other common pediatric solid tumors | 18/51 (35.29) | 3/12 (25.00) | 14/31 (45.16) | 0 | 1/8 (12.50) |
| **DCR** |  |  |  |  |  |
| Hodgkin lymphoma | 25/26 (96.15) | 15/15 (100.00) | 10/11 (90.91) | 0 | 0 |
| Non-Hodgkin lymphoma | 13/16 (81.25) | 3/3 (100.00) | 5/8 (62.50) | 5/5 (100.00) | 0 |
| Other common pediatric solid tumors | 38/51 (74.51) | 7/12 (58.33) | 25/31 (80.65) | 0 | 6/8 (75.00) |
| **Mean PFS (months)** |  |  |  |  |  |
| Hodgkin lymphoma | 38.8 (95% CI 24.7 - 53.0) | 46.8 (95% CI 30.7 - 63.0) | 24.8 (95% CI 13.0 - 36.6) | NA | NA |
| Non-Hodgkin lymphoma | 20.6 (95% CI 12.1 - 29.1) | NA | 19.8 (95% CI 8.3 - 31.4) | 15.2 (95% CI 11.0 - 19.4) | NA |
| Other common pediatric solid tumors | 12.6 (95% CI 8.0 - 17.1) | 13.0 (95% CI 4.7 - 21.3) | 10.6 (95% CI 7.2 - 14.1) | NA | 6.8 (95% CI 3.3 - 10.3) |
| **Median PFS (months)** |  |  |  |  |  |
| Hodgkin lymphoma | 35.6 (95% CI 9.3 - 62.0) | NA | 35.6 | NA | NA |
| Non-Hodgkin lymphoma | 17.6 | NA | NA | 17.6 (95% CI 0.2 - 34.9) | NA |
| Other common pediatric solid tumors | 8.6 (95% CI 3.0 - 14.3) | 2.7 (95% CI 0.0 - 7.6) | 12.1 (95% CI 6.2 - 18.1) | NA | 3.7 (95% CI 0.0 - 7.9) |
| **Mean DOR (months)** |  |  |  |  |  |
| Hodgkin lymphoma | 37.3 (95% CI 22.2 - 52.5) | 41.2 (95% CI 23.7 - 58.7) | 26.4 (95% CI 14.4 - 38.3) | NA | NA |
| Non-Hodgkin lymphoma | 23.8 (95% CI 14.7 - 32.8) | NA | NA | 12.9 (95% CI 8.7 - 17.1) | NA |
| Other common pediatric solid tumors | 19.4 (95% CI 11.9 - 26.9) | 19.7 (95% CI 5.0 - 34.4) | 14.0 (95% CI 9.1 - 18.9) | NA | 1.8 (95% CI 1.8 – 1.8) |
| **Median DOR (months)** |  |  |  |  |  |
| Hodgkin lymphoma | 31.2 | NA | 31.2 | NA | NA |
| Non-Hodgkin lymphoma | NA | NA | NA | 14.9 (95% CI 0.0 - 31.7) | NA |
| Other common pediatric solid tumors | NA | NA | 12.6 (95% CI 3.8 - 21.3) | NA | 1.8 |

Abbreviations: PD-1, programmed cell death receptor 1; HDACi, histone deacetylase inhibitor; VEGFR-TKI, vascular endothelial growth factor receptor tyrosine kinase inhibitor; ORR, objective response rate; DCR, disease control rate; PFS, progression free survival; NA, not achieved.
